# A Case of Forehead Subcutaneous Fat Atrophy Following Botulinum Toxin Injection

**DOI:** 10.1111/jocd.70140

**Published:** 2025-03-26

**Authors:** Wen Xu, Lijun He, Yeqin Dai

**Affiliations:** ^1^ Department of Dermatology Hangzhou Third People's Hospital, Hangzhou Third Hospital Affiliated to Zhejiang Chinese Medical University Hangzhou People's Republic of China; ^2^ School of Medicine Zhejiang University Hangzhou People's Republic of China; ^3^ Department of Ultrasound Hangzhou Third People's Hospital, Hangzhou Third Hospital Affiliated to Zhejiang Chinese Medical University Hangzhou People's Republic of China

To the Editor,

Botulinum toxin (BTX) is a cornerstone of minimally invasive cosmetic procedures, widely employed for dynamic wrinkle treatment due to its efficacy and favorable safety profile [1]. However, complications, though rare, can significantly affect patients both clinically and psychologically. Here, we report a case of forehead subcutaneous fat atrophy following BTX injection in an Asian female, shedding light on a rare complication and emphasizing the importance of precision in dosing and injection technique.

A 40‐year‐old Asian female developed progressive left forehead concavity 8 weeks post‐BOTOX (onabotulinumtoxinA, Allergan) treatment (Figure 1). Initial therapy delivered 2U (0.05 mL/site) of BTX‐A (100U/2.5 mL saline; 4U/0.1 mL) across five standard forehead sites. At 1‐week follow‐up for transient left frontal protrusions, she received a targeted 3U booster injection (0.075 mL) exclusively at the original left frontal site. The 1.2 × 1.4 cm paramedian depression emerged at 2 months, correlating with the secondary injection locus. Forehead kinematics remained intact with no neurologic sequelae. No interim interventions were reported.

**FIGURE 1:**
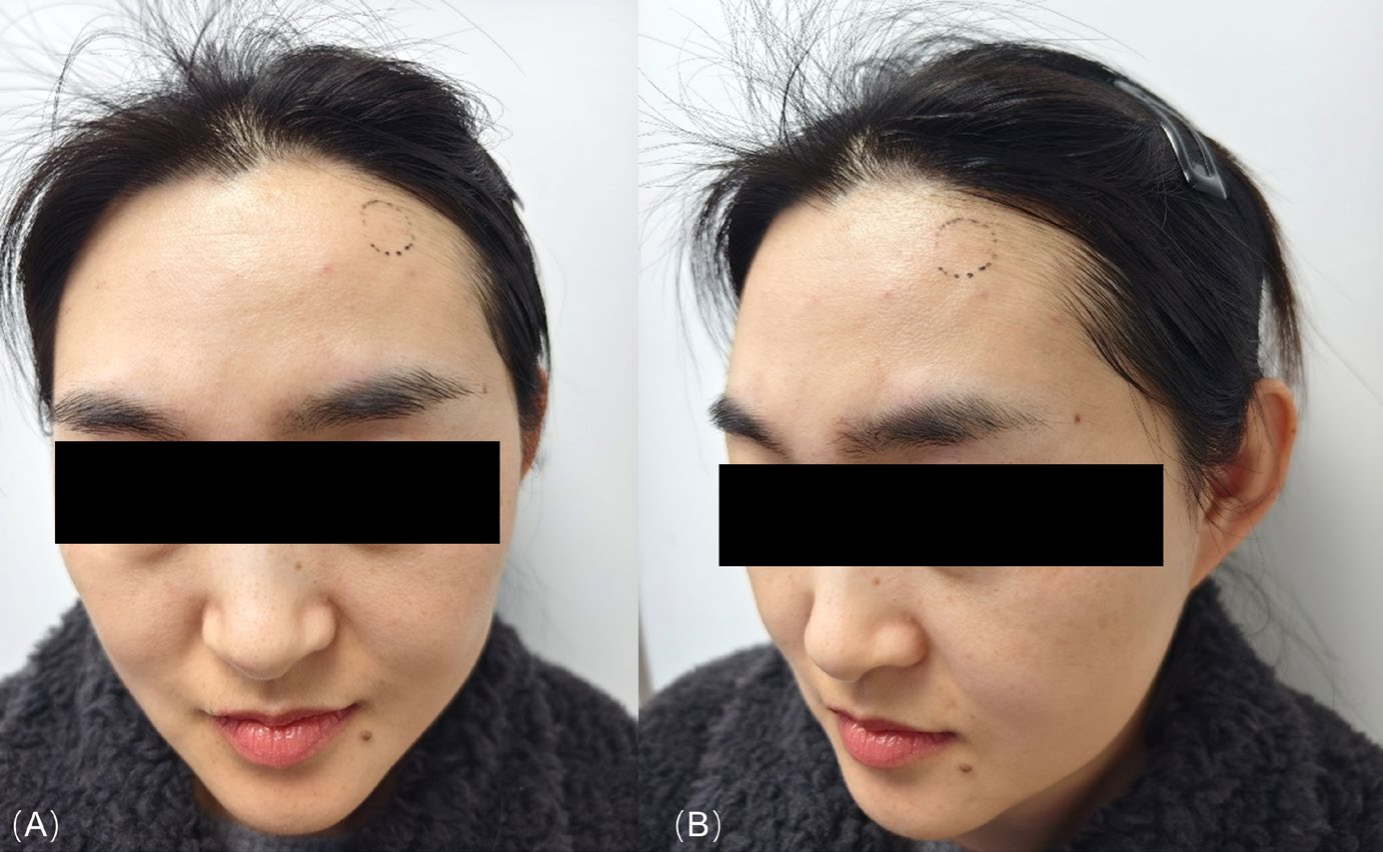
Frontal (A) and lateral (B) views of the patient's forehead showing a noticeable concavity in the left forehead region without skin discoloration or systemic involvement.

Ultrasound imaging revealed significant alterations in tissue structure, with reduced subcutaneous fat thickness (0.8 mm vs. 2.0 mm in the unaffected region) and thickening of the dermis (1.8 mm vs. 1.4 mm) and frontalis muscle, and the subfrontal space (0.9 mm vs. 0.4 mm) (Figure 2). These findings confirmed localized atrophy and structural changes in the affected area.

**FIGURE 2:**
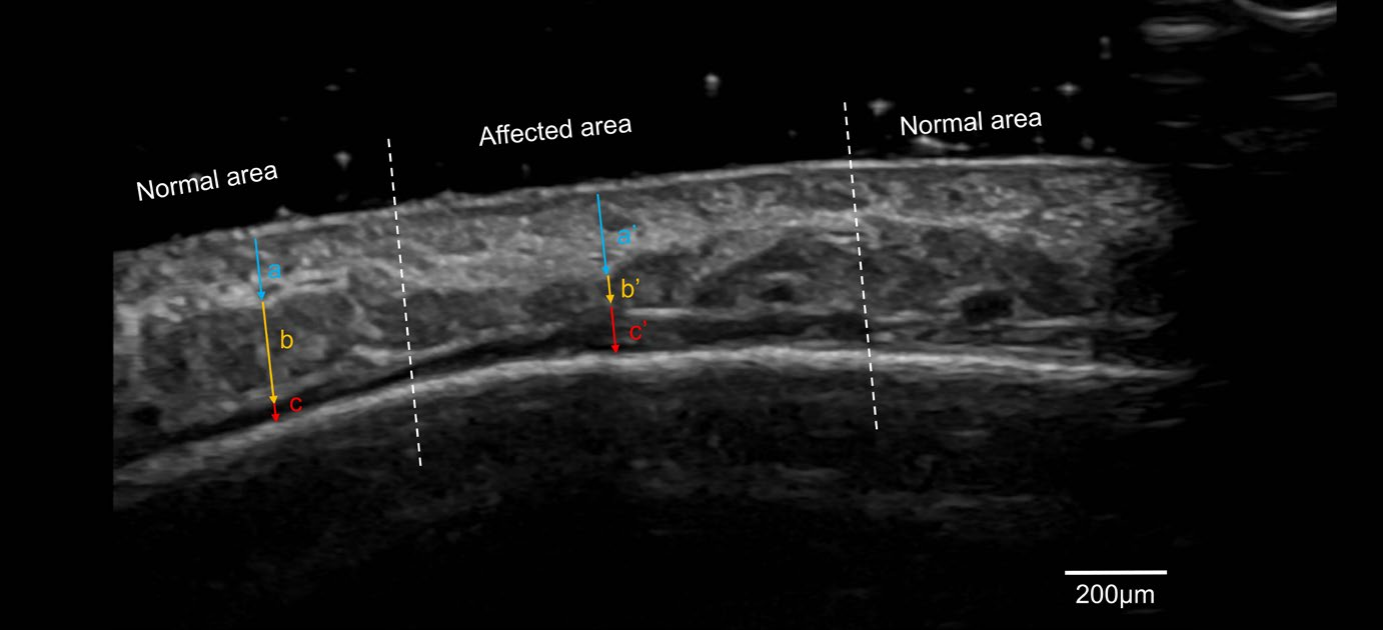
Ultrasound images of the patient's forehead, showing both normal and affected regions. (a) and (a’) correspond to the dermal thickness in the normal and affected regions, measuring 1.4 and 1.8 mm, respectively. (b) and (b’) represent the thickness of the subcutaneous fat layer in the normal and affected regions, measuring 2.0 and 0.8 mm, respectively. (c) and (c’) depict the thickness of the frontalis muscle and the subfascial space in the normal and affected regions, measuring 0.4 and 0.9 mm, respectively.

Fat atrophy remains uncommon in aesthetic BTX applications at therapeutic doses. This case highlights critical considerations for patients with anatomical predispositions, particularly the thinner subcutaneous fat layers prevalent in Asian populations. The localized nature of atrophy—without systemic symptoms or bilateral involvement—excludes metabolic etiologies, reinforcing localized neuro‐adipose disruption as the likely mechanism.

The observed fat loss aligns with Lim & Seet's hypothesis of chemodenervation‐induced lipolysis via BTX diffusion into adipose innervation [2]. This contrasts sharply with BTX's protective role in fat graft survival through mechanical stress reduction [3], and differs from Fortuna et al.'s chronic intramuscular fat replacement in denervated muscles [4]. Although intestinal BTX accelerates gut transit to reduce fat absorption systemically [5], this case aligns with anatomical stratification of BTX effects: superficial neuro‐adipose disruption precipitating lipolysis versus deeper mechanical protection preserving fat viability, underscoring the critical role of injection depth in predisposed populations.

In conclusion, this case highlights the necessity of precision in dosing and technique to minimize complications. Clinicians should maintain a high index of suspicion for rare adverse effects, particularly in demographics with unique anatomical characteristics. Further research is warranted to explore mechanisms underlying localized fat atrophy following BTX and to establish preventative strategies.

## Author Contributions

**Wen Xu:** writing – original draft. **Lijun He:** validation, supervision. **Yeqin Dai:** writing – review and editing, supervision.

## Consent

The patient provided written consent to publish her case details and accompanying photographs.

## Conflicts of Interest

The authors declare no conflicts of interest.

## Data Availability

Data sharing not applicable to this article as no datasets were generated or analysed during the current study.
